# The correlation of atherosclerosis and triglyceride glucose index: a secondary analysis of a national cross-sectional study of Japanese

**DOI:** 10.1186/s12872-022-02685-8

**Published:** 2022-06-03

**Authors:** Xingping Yang, Zhao Gao, Xuming Huang, Mingxing Zhang, Zhuoming Chen

**Affiliations:** 1 Department of Rehabilitation Medicine, The First Affiliated Hospital, Jinan University, No. 613 West Huangpu Avenue, Tianhe District, Guangzhou, 510105 China; 2 Department of Rehabilitation Medicine, The First Affiliated Hospital of Guangdong Pharmaceutical University, Guangzhou, 510080 China; 3 Office of Academic Research, Er Sha Sports Training Center of Guangdong Province, Guangzhou, 510105 China

**Keywords:** Triglyceride glucose index, Brachial-ankle pulse wave velocity, Atherosclerosis, Cardiovascular diseases, Japanese, Insulin resistance

## Abstract

**Background:**

Few studies examined the relationship between triglyceride/glucose index (TyG index) and atherosclerosis in Japanese adults. Therefore, this study evaluated their relationship, as measured based on the brachial-ankle pulse wave velocity (baPWV) in Japanese adults.

**Methods:**

A total of 912 participants was selected from the NAGALA (NAFLD in Gifu Area, Longitudinal Analysis) study conducted from 2004 to 2012. The relationship between the TyG index and baPWV was estimated through a logistic model. Subgroup analyses by sex, age, body mass index (BMI), total cholesterol, low-density lipoprotein cholesterol, estimated glomerular filtration rate (eGFR), and fatty liver was performed. The formula for TyG index was ln (½fasting triglyceride level [mg/dL] × fasting plasma glucose level [mg/dL]).

**Results:**

A linear relationship between TyG and baPWV was discovered after adjusting for underlying confounders. An increased risk of baPWV was observed after adjusting for sex, age, BMI, systolic blood pressure, diastolic blood pressure, high-density lipoprotein cholesterol, fatty liver, eGFR, and TyG as a continuous variable (adjusted odds ratio [adj OR], 1.57; 95% confidence interval [95% CI], 1.14–2.18). Compared with the TyG index in the first tertile, the probabilities of subjects in the third tertile that developed to baPWV were 1.78-fold higher (adj OR 1.78, 95% CI 1.08–2.95: P for trend 0.024). Moreover, stable associations were observed between the TyG index and baPWV in different variables through subgroup analyses.

**Conclusions:**

The highest tertile (above 8.57) of the TyG index was positively and linearly related to subclinical atherosclerosis in Japanese adults and may be valuable as a predicted marker.

## Background

Cardiovascular diseases (CVDs) are the leading cause of death worldwide [1]. CVDs comprise peripheral and coronary artery diseases, cardiac valve disease, cardiac muscle disorder, congenital heart disease to arrhythmias, and ultimately, heart failure [2]. CVDs are associated with vulnerable atherosclerotic plaques [3], formed because of increased luminal pressure and shear stress due to arterial stiffening, causing the acceleration of atheroma formation, stimulation of excessive collagen production, and deposition in the arterial wall, leading to atherosclerosis [4, 5]. Thus, according to a recent study, the brachial-ankle pulse wave velocity (baPWV), a candidate measure of subclinical atherosclerosis, can independently predict cardiovascular risk, representing a high-priority therapeutic target to ameliorate CVD [6].

Insulin resistance (IR) patients are vulnerable to the development of accelerated atherosclerosis [7]. Recently, studies have demonstrated that the TyG index, which is calculated as ln [fasting triglyceride (mg/dL) × fasting glucose (mg/dL)/2], was adopted to evaluate IR with 84.0% sensitivity and 45.0% specificity [8]. Thus, the TyG index could serve as a simple and credible surrogate marker of IR [9, 10]. In clinical practice, BaPWV is a simple and reliable tool to measure atherosclerosis due to its high reproducibility [11–15]. However, the relationship between the TyG index and baPWV is uncertain and might vary among populations [16–20], with studies showing relationships between TyG and baPWV in Chinese [16, 18]. The relationship between TyG and baPWV can be affected by various factors like smoking, alcohol consumption, and fatty liver [17].

Although the TyG index has been suggested as a reliable IR marker, the relation between the TyG index and atherosclerosis in a Japanese population is yet to be determined. In this study, we evaluated the relationship between the TyG index and atherosclerosis based on the baPWV in Japanese adults. The research will provide an insight into the role of IR in atherosclerosis.

## Methods

### Data source

The secondary use of the datasets was explored through the DATADRYAD database (http://www.Datadryad.org/), which provides free access to the original research data (Fukuda Takuya et al. (2014)) (dataset: https://doi.org/10.5061/dryad.m484p). The database provided variables including sex, age, body mass index (BMI), diastolic blood pressure (DBP), systolic blood pressure (SBP), alanine aminotransferase (ALT), aspartate aminotransferase (AST), gamma-glutamyl transferase (γGTP), fasting plasma glucose (FPG), uric acid (UA), total cholesterol (TC), triglyceride (TG), high-density lipoprotein cholesterol (HDL-C), low-density lipoprotein cholesterol (LDL-C), estimated glomerular filtration rate (eGFR), ankle-brachial index (ABI), brachial-ankle pulse wave velocity (baPWV).

### Examinations

In the original study, the routine tests included urinalysis, blood cell counts, blood chemistry, hepatitis B antigen, hepatitis C antibody, ECG, chest radiography, barium examination of the upper gastrointestinal tract, and abdominal ultrasonography. The 8-h fasting blood samples were drawn from the antecubital vein of seated participants. The blood samples were collected in siliconized glass tubes containing sodium fluoride for the glucose analysis and no additives for the serum. Plasma and serum samples were obtained by centrifugation immediately after sampling and stored at − 80 °C. Blood tests were performed using a MODULAR ANALYTICS (Hitachi High-Technologies Corp. Ltd, Tokyo, Japan). The coefficients of variation for γGTP, ALT, AST, plasma glucose, triglycerides, HDL-C, LDL-C, UA, and creatinine were 2%, 1.9%, 1.7%, 1.2%, 2.3%, 2.1%, 2.5%, 0.9% and 1.4%, respectively.

Abdominal ultrasound was performed for the diagnosis of the fatty liver using an Aloka SSD-650CL (Aloka Co, Ltd, Tokyo, Japan). BMI was calculated as kg/m^2^.

### Study population

**Fig. 1:**
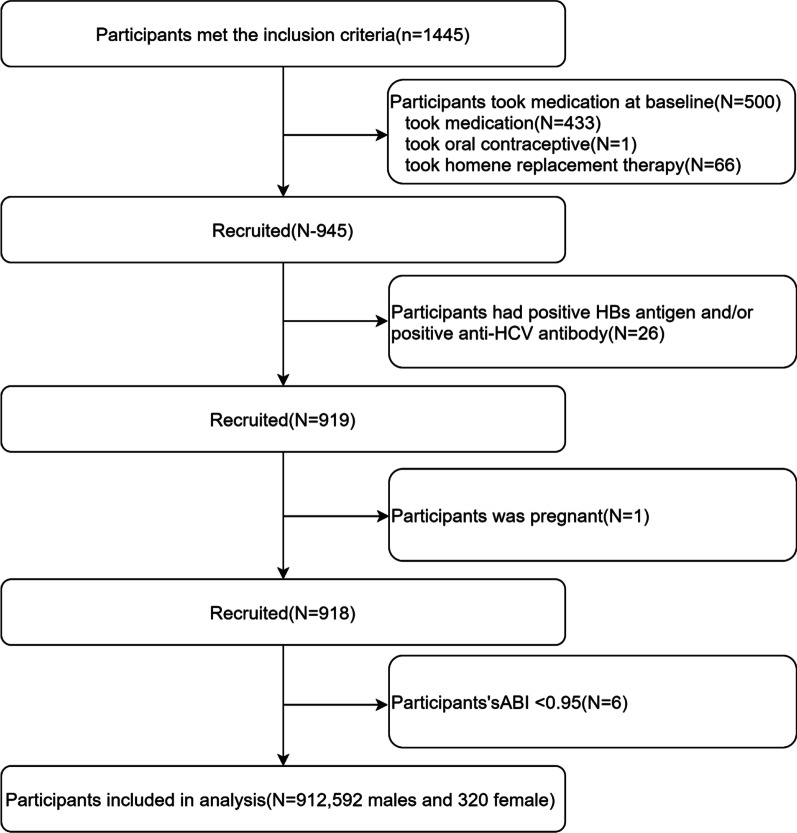
Flow diagram of the screening and enrollment of study participants. HBs, hepatitis B surface; HCV, hepatitis C virus; ABI, ankle-brachial index

A physical examination research project from the Murakami Memorial Hospital (Gifu, Japan) was performed. The aim was to detect chronic diseases early and evaluate their underlying risk factors to improve public health. With the database, Fukuda et al. [21] reported that serum γGTP affected the risk of developing atherosclerosis in women. The participants underwent an examination between March 2004 and December 2012. A total of 1445 participants (897 men and 548 women) was chosen based on the following exclusion criteria: (1) taking exogenous hormone supplementation at baseline; (2) hepatitis B (HBV) or C virus (HCV) infection; (3) fetation; (4) ABI < 0.95. A total of 912 participants (592 men and 320 women) was included in the complete analysis (Fig. 1). The study was approved by the ethics committee of the Murakami Memorial Hospital, and all participants provided informed consent before entering the research project.

### Data collection and measurements

The database contains information on participants' demographic characteristics, biochemical indices, abdominal ultrasonography, baPWV, ABI, and lifestyle factors. During the latest month, the average weekly alcohol consumption was recorded and classified into < 40, 40–140, 140–280, and > 280 g/week, respectively [22]. The participants were divided into never, former, and current smokers. Exercise that caused sweating, such as jogging, cycling, and swimming, was perceived to be normal for more than a week [23]. Abdominal ultrasound, together with the diagnosis by gastroenterologists, confirmed participants were suffering from fatty liver. Among the four criteria (liver/kidney echo contrast, liver luminance, deep attenuation, and vascular obscurity), participants with liver brightness and liver/kidney contrast were considered fatty liver disease patients [24]. The baPWV and ABI of patients were measured using an automatic waveform analyzer (Colin Medical Technology, Komaki, Japan) at room temperature [21]. The path length from the suprasternal notch to the humerus (Lb) and the ankle joint (La) was determined according to the subject's height. The delay time from the ascending point of the brachial waveform to the ascending point of the ankle waveform (DTba) was automatically determined. The incidence of baPWV was assessed with the pulse wave propagation distance (Lb-La) modified by the pulse wave propagation time (DTba) divided into cm/s [25]. The reported inter-observer and its coefficients of variation were 10% (r = 0.87, *p* < 0.01) and 8.4% (r = 0.98, *p* < 0.01), respectively. The eGFR for man was evaluated using the formula as shown in Eq. 1 and was multiplied by a correction factor of 0.739 for women [26].

1 $${\text{eGFR}}\, = \,{194}\, \times \,{\text{Cr}}\, - \,{1}.0{94}\, \times \,{\text{age}}\, - \,0.{287 }\left( {{\text{mL}}/{\text{min}}/{1}.{\text{73 m}}^{{2}} } \right).$$

The formula for the TyG index was as shown in Eq. 2 [8].

2 $${\text{ln }}{\raise0.5ex\hbox{$\scriptstyle 1$} \kern-0.1em/\kern-0.15em \lower0.25ex\hbox{$\scriptstyle 2$}}{\text{fasting triglyceride level }}\left( {{\text{mg}}/{\text{dL}}} \right)\, \times \,{\text{fasting plasma glucose }}\left( {{\text{FPG}}} \right) \, \left( {{\text{mg}}/{\text{dL}}} \right)$$

### Statistical analysis

Normally and skewed distributed continuous variables were expressed as means ± standard deviation (SD) and medians with inter-quaternary interval (IQRS). Categorical variables were expressed in terms of frequency and percentage. This study used the chi-square test, one-way analysis of variance, or Kruskal–Wallis test to examine the statistical significance of the differences between groups stratified by the tertiles of the TyG index. Univariable and multivariable logistic proportional hazard model was used to examine the relationship between the TyG index and the risk of baPWV. Three models were employed: model 1 as a crude model: univariable; model 2 modified with age and gender; and model 3, adjusted for model 2 plus BMI, SBP, DBP, HDL-C, fatty liver, and eGFR. In these models, the median values in each tertile of the TyG index were used to operate linear trend tests. The logistic relationship between the TyG and baPWV was estimated using logistic regression with restricted cubic splines. For identification of modifications and interactions, subgroups analyses were developed with stratified linear regression models and the likelihood ratio test (LRT) regarding sex, age (< 55 or ≥ 55 years), BMI (< 25 or ≥ 25 kg/m^2^), TC (< 208 or ≥ 208 mg/dL), LDL-C (< 120.1 or ≥ 120.1 mg/dL) [27], eGFR (< 60 or ≥ 60 mL/min/1.73 m^2^), and fatty liver (none, moderate, or severe). All analyses were performed using the statistical software packages in R 3.3.2 (http://www.R-project.org, The R Foundation) and Free Statistics software version 1.1. Differences with *p*-values < 0.05 were considered statistically significant.

## Results

### Baseline characteristics of selected participants

In this cross-sectional study, 912 Japanese participants were included. The mean age was 51.1 ± 9.6 years, and 64.9% were male. Grouping was made according to the TyG index of the participants, which are the first group of TyG (Q1), the second group of TyG (Q2), and the third group of TyG (Q3). The baseline characteristics of the participants according to the tertiles of the TyG index are shown in Table 1. Participants in the third group of TyG (Q3) were more likely to be male and had higher TyG values. They had higher BMI, SBP, DBP, FBG, uric acid, AST, ALT, γGTP, TC, TG, and LDL-C levels, and higher eGFR. They were more likely to be current smokers, consume > 280 g/week of alcohol, exercise < 1/week, have moderate or severe fatty liver, and have a higher baPWV than the other groups (Q1–2). The distribution of male sex was 42.1% in Q1, 66.8% in Q2, and 85.9% in Q3. Participants in the third group of TyG (Q3) had lower HDL-C and eGFR values and were more likely to be none or past smokers, consume 0–40 g/week alcohol, exercise ≥ 1/week, and have no fatty liver compared to the other groups (Q1–2).

**Table 1:** Baseline characteristics of the study participants sorted by tertiles of TyG

	All participants	Q1 (< 7.96)	Q2 (7.96–8.57)	Q3 (> 8.57)	*p* value
Participants (n)	912	304	304	304	
TyG index	8.3 ± 0.7	7.6 ± 0.3	8.3 ± 0.2	9.0 ± 0.4	< 0.001***
Sex (n%)					< 0.001***
Males	592 (64.9)	128 (42.1)	203 (66.8)	261 (85.9)	
Females	320 (35.1)	176 (57.9)	101 (33.2)	43 (14.1)	
Age (years)	51.1 ± 9.6	50.7 ± 10.1	51.6 ± 9.4	51.1 ± 9.2	0.493
BMI (kg/m^2^)	23.1 ± 3.1	21.8 ± 2.5	23.1 ± 3.3	24.5 ± 3.0	< 0.001***
SBP (mmHg)	120.2 ± 15.0	114.7 ± 13.9	120.9 ± 15.4	125.1 ± 13.7	< 0.001***
DBP (mmHg)	76.1 ± 10.0	72.0 ± 9.4	76.4 ± 10.2	80.0 ± 8.7	< 0.001***
FBG (mg/dL)	98.1 ± 14.1	92.2 ± 7.6	98.0 ± 10.4	104.0 ± 18.9	< 0.001***
Uric acid (mg/dL)	5.3 ± 1.4	4.6 ± 1.3	5.2 ± 1.3	6.0 ± 1.3	< 0.001***
AST (IU/L)	19.0 (16.0, 23.0)	19.0 (16.0, 22.0)	19.0 (16.0, 23.0)	21.0 (17.0, 26.0)	< 0.001***
ALT (IU/L)	19.0 (14.0, 26.0)	16.0 (12.8, 20.0)	18.0 (14.0, 23.2)	23.0 (18.0, 34.0)	< 0.001***
γGTP (IU/L)	19.0 (14.0, 28.0)	14.0 (11.0, 19.0)	18.5 (14.0, 26.0)	25.0 (18.0, 43.0)	< 0.001***
TC (mg/dL)	209.8 ± 35.9	198.4 ± 33.9	208.0 ± 33.7	223.1 ± 35.8	< 0.001***
TG (mg/dL)	99.9 ± 74.9	44.2 ± 12.3	81.8 ± 14.8	173.6 ± 87.3	< 0.001***
HDL-C (mg/dL)	53.5 ± 14.6	62.0 ± 14.0	53.4 ± 13.1	45.2 ± 11.4	< 0.001***
LDL-C (mg/dL)	128.1 ± 31.7	116.6 ± 29.2	130.4 ± 28.7	137.1 ± 33.5	< 0.001***
eGFR (mL/min/1.73 m^2^)	70.4 ± 12.0	73.5 ± 13.0	70.2 ± 11.4	67.6 ± 11.0	< 0.001***
Current smoker (n%)					< 0.001***
None or past	715 (78.4)	263 (86.5)	233 (76.6)	219 (72)	
Current	197 (21.6)	41 (13.5)	71 (23.4)	85 (28)	
Alcohol group (n%)					< 0.001***
0–40 g/week	594 (65.1)	225 (74)	194 (63.8)	175 (57.6)	
40–140 g/week	150 (16.4)	42 (13.8)	59 (19.4)	49 (16.1)	
140–280 g/week	88 (9.6)	23 (7.6)	33 (10.9)	32 (10.5)	
> 280 g/week	80 (8.8)	14 (4.6)	18 (5.9)	48 (15.8)	
Regular exercise (n%)					0.008**
< 1/week	735 (80.6)	229 (75.3)	247 (81.2)	259 (85.2)	
≥ 1/week	177 (19.4)	75 (24.7)	57 (18.8)	45 (14.8)	
Fatty liver (n%)					< 0.001***
None	647 (70.9)	274 (90.1)	229 (75.3)	144 (47.4)	
Moderate or severe	265 (29.1)	30 (9.9)	75 (24.7)	160 (52.6)	
Menopausal state (n%)					0.071
Menopausal	138 (43.1)	86 (48.9)	36 (35.6)	16 (37.2)	
Postmenopausal	182 (56.9)	90 (51.1)	65 (64.4)	27 (62.8)	
ABI	1.2 ± 0.1	1.2 ± 0.1	1.2 ± 0.1	1.2 ± 0.1	0.001**
baPWV(cm/s)	1415.8 ± 246.3	1350.7 ± 226.1	1424.5 ± 234.6	1472.1 ± 262.0	< 0.001***

Values are expressed as mean ± standard deviation or n (%). Abbreviations: TyG index, triglyceride glucose index; BMI, body mass index; SBP, systolic blood pressure; DBP, diastolic blood pressure; FPG, fasting plasma glucose; AST, aspartate aminotransferase; ALT, alanine aminotransferase; γGTP, gamma-glutamyl transferase; TC, total cholesterol; TG, triglyceride; HDL-C, high-density lipoprotein cholesterol; LDL-C, low-density lipoprotein cholesterol; eGFR: estimated glomerular filtration rate; ABI, ankle-brachial index; baPWV, brachial to ankle pulse wave velocity. (**p < 0.01; ***p < 0.001)

### Univariable analysis for baPWV

As shown in Table 2, the relationship between risk factors and baPWV was assessed using univariable analyses. The results revealed that sex, age, SBP, DBP, FBG, uric acid, AST, ALT, γGTP, TC, TG, TyG, and LDL-C levels, eGFR, alcohol use, fatty liver, and menopausal state were positively related to baPWV (*p < 0.05; **p < 0.01; ***p < 0.001; 95%CI). However, the BMI, HDL-C level, current smoker, regular exercise, and ABI were not associated with baPWV (*p* > 0.05; 95%CI). These results suggest that in most instances, there are increased risks associated with baPWV.

**Table 2:** Univariable analysis of baPWV

	OR (95%CI)	*p* value
Sex (n%)		0.035*
Males	Ref.	
Females	0.74 (0.56,0.98)	
Age (year)	1.10 (1.09,1.12)	< 0.001***
BMI (kg/m^2^)	1.04 (1.00,1.08)	0.069
SBP (mmHg)	1.07 (1.06,1.09)	< 0.001***
DBP (mmHg	1.10 (1.09,1.12)	< 0.001***
FBG (mg/dL)	1.04 (1.02,1.05)	< 0.001***
Uric acid (mg/dL)	1.14 (1.04,1.26)	0.007**
AST (IU/L)	1.03 (1.01,1.04)	0.004**
ALT (IU/L)	1.01 (1.00,1.02)	0.024*
γGTP (IU/L)	1.01 (1.00,1.02)	0.001**
TC (mg/dL)	1.01(1.00,1.01)	< 0.001***
TG (mg/dL)	1.00 (1.00,1.01)	< 0.001***
TyG index	1.84 (1.50,2.27)	< 0.001***
HDL-C (mg/dL)	0.99 (0.99,1.00)	0.249
LDL-C (mg/dL)	1.01 (1.00,1.01)	0.012*
eGFR (mL/min/1.73 m^2^)	0.96 (0.94,0.97)	< 0.001***
Current smoker (n %)		0.467
None or past	Ref.	
Current	0.89 (0.65,1.22)	
Alcohol group (n %)		0.08
0–40 g/week	Ref.	
40–140 g/week	0.98 (0.68,1.40)	0.896
140–280 g/week	1.35 (0.86,2.11)	0.192
> 280 g/week	1.73 (1.08,2.77)	0.022*
Regular exercise (n %)		0.814
< 1/week	Ref.	
≥ 1/week	1.04 (0.75,1.45)	
Fatty liver (n %)		< 0.001***
None	Ref.	
Moderate or severe	1.92 (1.44,2.56)	
Menopausal state (n %)		< 0.001***
Menopausal	Ref.	
Postmenopausal	5.37 (3.20,9.01)	< 0.001***
ABI	3.20 (0.49,20.72)	0.222

BMI, Body mass index; SBP, systolic blood pressure; DBP, diastolic blood pressure; FPG, fasting plasma glucose; AST, aspartate aminotransferase; ALT, alanine aminotransferase; γGTP, gamma-glutamyl transferase; TC, total cholesterol; TG, triglyceride; TyG index, triglyceride glucose index; HDL-C, high-density lipoprotein cholesterol; LDL-C, low-density lipoprotein cholesterol; eGFR, estimated glomerular filtration rate; ABI, ankle-brachial index; baPWV, brachial to ankle pulse wave velocity; CI, confidence interval. (*p < 0.05; **p < 0.01; ***p < 0.001)

### Unadjusted and adjusted logistics models

Linear logistic models were used to evaluate the independent relationship between the TyG index and baPWV (univariable and multivariable logistic models). Table 3 presents the effect sizes (ORs) and 95% confidence intervals (95% CIs). In the crude model (model 1), a one-unit increase in the TyG index was related to an 84% higher risk of incident increased baPWV (OR 1.84, 95% CI 1.50–2.27). In model 2, a one-unit increase in the TyG index increased the risk of evolving baPWV by 91% (OR 1.91, 95% CI 1.49–2.44) after adjusting for gender and age. In model 3, each one-unit increase in the TyG index was a 57% higher risk of incident increased baPWV (OR 1.57, 95% CI 1.14–2.18). The study transformed the TyG index into a categorical variable (tertile of the TyG index). The results showed that the ***P***-value for the trend of the TyG index as categorical variables was concomitant with the result of the TyG index as continuous variables in the different models. Moreover, in model 3, the highest tertile (above 8.57) of the TyG index was statistically significant with baPWV.

**Table 3:** Relationship between TyG and baPWV in different models

	Model 1	*p* value	Model 2	*p* value	Model 3	*p* value
TyG index	1.84 (1.50,2.27)	< 0.001	1.91 (1.49,2.44)	< 0.001	1.57 (1.14,2.18)	0.006**
TyG index						
Q1 (< 7.96)	Ref.		Ref.		Ref.	
Q2 (7.96–8.57)	1.74 (1.25,2.42)	< 0.001	1.66 (1.15,2.41)	0.196	1.43 (0.93,2.19)	0.101
Q3 (> 8.57)	2.42 (1.74,3.36)	< 0.001	2.50 (1.70,3.71)	< 0.001	1.78 (1.08,2.95)	0.024*
*p* for trend	< 0.001		< 0.001		0.024	

Model 1 was not adjusted. Model 2 was adjusted for sex and age. Model 3 was adjusted for sex, age, BMI, SBP, DBP, HDL-C, fatty liver, eGFR. BMI, body mass index; SBP, systolic blood pressure; DBP, diastolic blood pressure; HDL-C, high-density lipoprotein cholesterol; eGFR, estimated glomerular filtration rate; TyG, triglyceride glucose index; baPWV, brachial to ankle pulse wave velocity; CI, confidence interval. (*p < 0.05; **p < 0.01)

### Threshold effect analysis of the TyG on incident baPWV

**Fig. 2:**
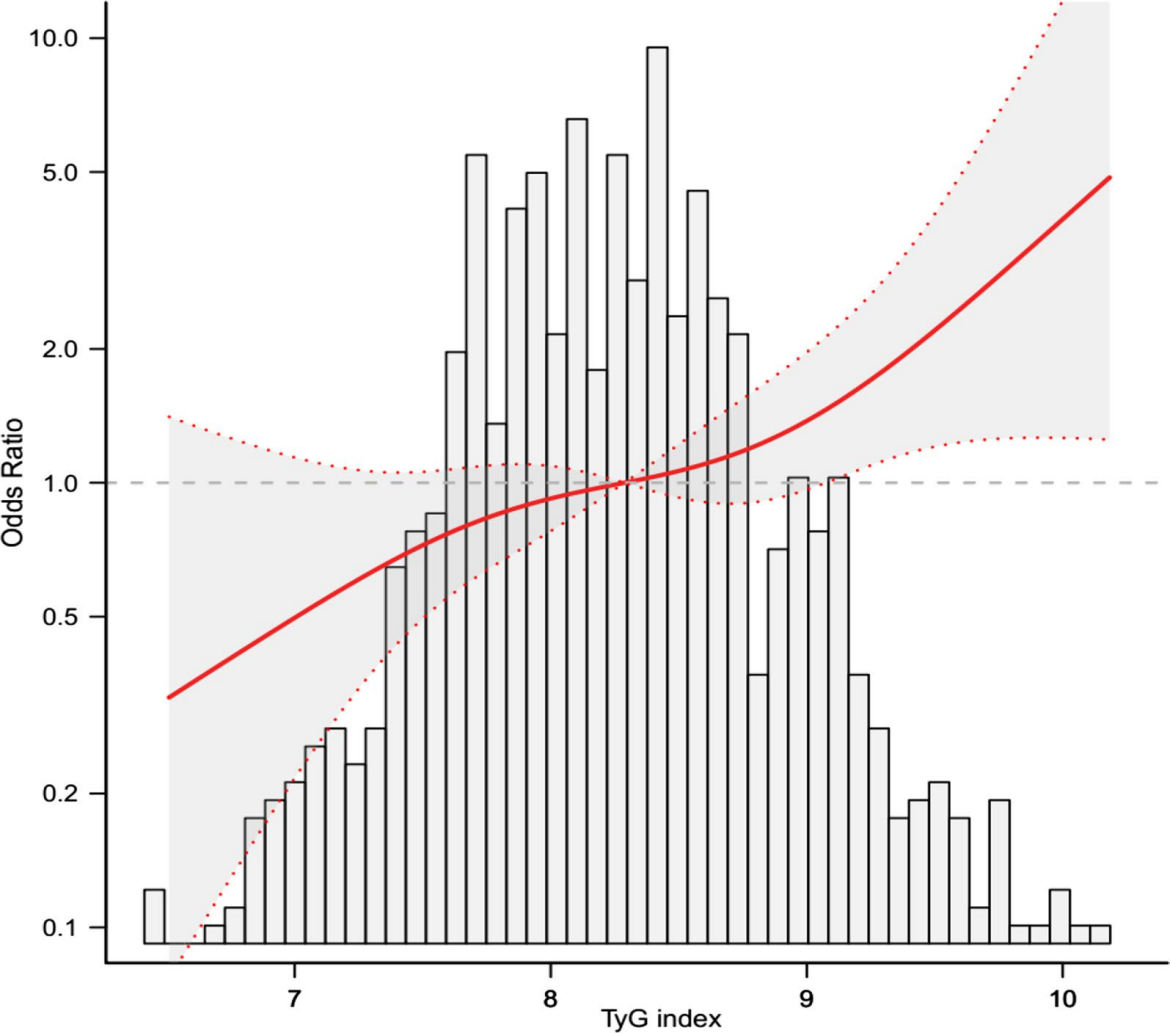
The relations between the TyG index and baPWV. The grey histograms represent the distributions between variables, and the solid red curve indicates the smoothing curve fit between variables. The grey zone indicates the 95% confidence interval of the curve fit. Data were adjusted for gender, age, BMI, SBP, DBP, HDL-C, fatty liver, eGFR, and a positive linear correlation can be seen between the TyG index and baPWV. BMI, body mass index; SBP, systolic blood pressure; DBP, diastolic blood pressure; HDL-C, High-density lipoprotein cholesterol; eGFR, estimated glomerular filtration rate; TyG, triglyceride glucose index; baPWV, brachial to ankle pulse wave velocity; CI, confidence interval

This study used a logistic regression model with a cubic spline function to assess the correlation between the TyG index and baPWV. The distributions between variables (grey histograms), smoothing curve fit between variables (solid red curve), and 95% confidence interval of the curve fit (grey zone) are shown in Fig. 2. After adjusting for sex, age, BMI, SBP, DBP, TC, LDL-C, fatty liver, and eGFR, the highest tertile (above 8.57) of the TyG index was linearly associated with the baPWV.

### Subgroup analyses

**Fig. 3:**
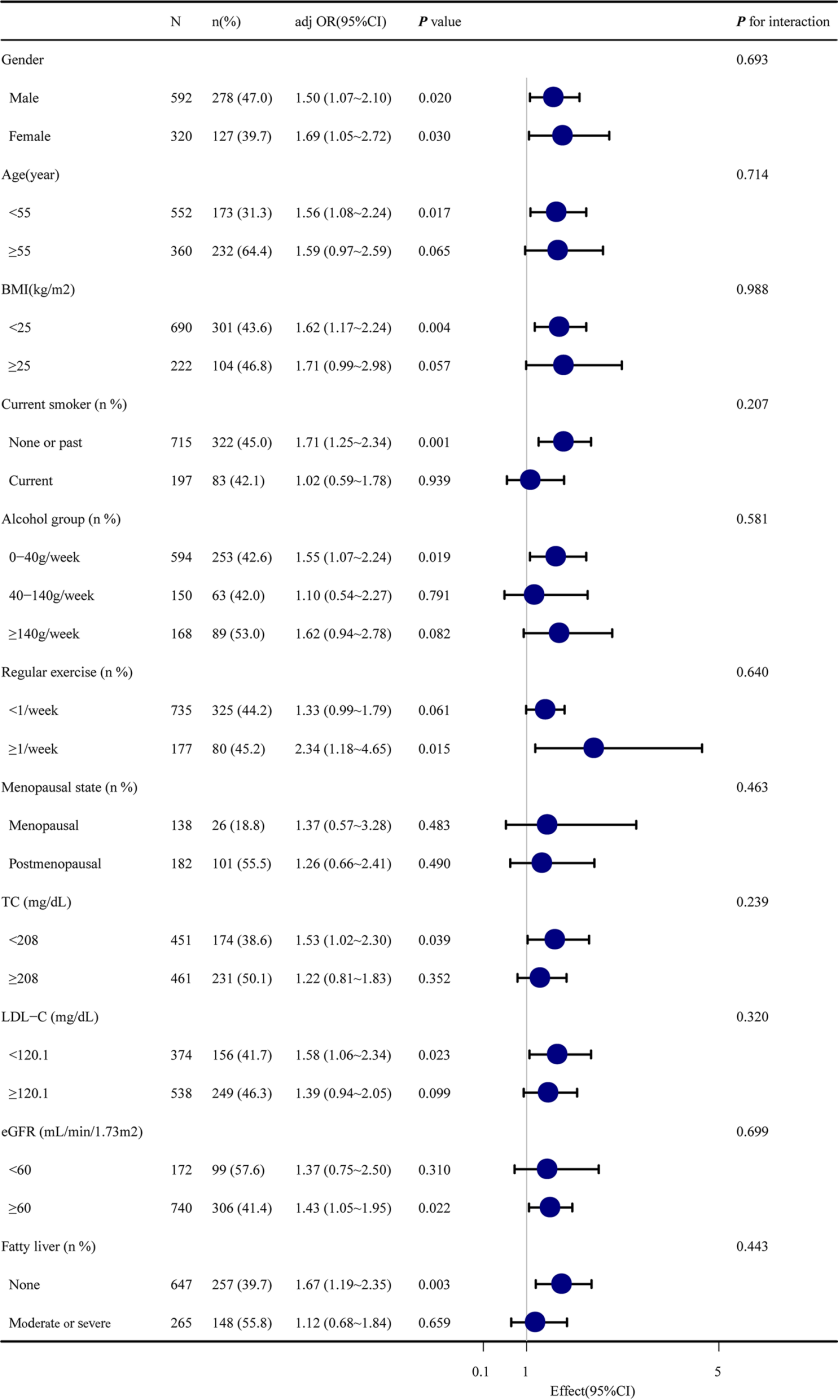
Subgroup analysis of the association between TyG index and baPWV.The participants have split into subgroups accordingly and adjusted for gender, age, BMI, SBP, DBP, HDL-C, fatty liver, and eGFR. A steady relationship can be seen between the TyG index and baPWV in different subgroups. BMI, body mass index; SBP, systolic blood pressure; DBP, diastolic blood pressure; HDL-C, high-density lipoprotein cholesterol; eGFR, estimated glomerular filtration rate; TyG, triglyceride glucose index; baPWV, brachial to ankle pulse wave velocity; CI, confidence interval. (*p < 0.05; **p < 0.01; ***p < 0.001)

The results of subgroup analyses of the association between the TyG index and baPWV are shown in Fig. 3 and Table 4. The participants were split into subgroups according to and adjusted for sex, age, BMI, SBP, DBP, HDL-C, fatty liver, and eGFR. The results revealed that sex (both male and female), BMI (less than 25 kg/m^2^), age (less than 55 years), TC (less than 208 mg/dL), LDL-C (less than 120.1 mg/dL), eGFR (more than or equal 60 mL/min/1.73m^2^), smoking (none or past), alcohol (0–40 g/week), exercise (≤ 1/week), and not having fatty liver disease (647 participants) were significant (all *p* < 0.05). These results suggested a steady relationship between TyG and the incident of baPWV in different subgroups. This clearly means that the TyG index was independently and steadily associated with incident baPWV. However, there was no significant interaction between the TyG index and incident baPWV in any subgroup analysis results.

**Table 4:** Subgroup analyses of the association between TyG index and baPWV

Subgroup	N	N (%)	adj OR (95%CI)	*p* value	*p* for interaction
*Sex*					
Male	592	278 (47)	1.71 (1.14–2.57)	0.009	0.891
Female	320	127 (39.7)	0.98 (0.54–1.78)	0.958	
*Age (years)*					
< 55	552	173 (31.3)	1.38 (0.88–2.16)	0.165	0.719
≥ 55	360	232 (64.4)	2 (1.11–3.63)	0.022	
*BMI (kg/m*^*2*^*)*					
< 25	690	301 (43.6)	1.39 (0.95–2.03)	0.094	0.952
≥ 25	222	104 (46.8)	1.94 (0.95–3.93)	0.067	
*Current smoker (n%)*					
None or past	715	322 (45)	1.58 (1.07–2.31)	0.02	0.219
Current	197	83 (42.1)	1.07 (0.55–2.09)	0.834	
*Alcohol group (n%)*					
0–40 g/week	594	253 (42.6)	1.38 (0.88–2.17)	0.159	0.162
40–140 g/week	150	63 (42)	1.15 (0.48–2.77)	0.76	
140–280 g/week	88	44 (50)	5.62 (1.41–22.51)	0.015	
> 280 g/week	80	45 (56.2)	1.27 (0.47–3.41)	0.641	
*Regular exercise (n%)*					
< 1/week	735	325 (44.2)	1.25 (0.87–1.79)	0.23	0.923
≥ 1/week	177	80 (45.2)	3.28 (1.28–8.39)	0.013	
*Fatty liver (n%)*					
None	647	257 (39.7)	1.37 (0.92–2.05)	0.12	0.569
Moderate or severe	265	148 (55.8)	1.43 (0.77–2.64)	0.257	
*Menopausal state (n%)*					
Menopausal	138	26 (18.8)	0.97 (0.29–3.24)	0.958	0.554
Postmenopausal	182	101 (55.5)	1.03 (0.46–2.3)	0.951	
*LDL-C (mg/dL)*					
< 120.1	374	156 (41.7)	1.31 (0.8–2.15)	0.279	0.355
≥ 120.1	538	249 (46.3)	1.29 (0.81–2.08)	0.286	
*eGFR (mL/min/1.73 m*^*2*^*)*					
< 60	172	99 (57.6)	1.59 (0.78–3.23)	0.202	0.531
≥ 60	740	306 (41.4)	1.27 (0.87–1.85)	0.219	

BMI, Body mass index; SBP, systolic blood pressure; DBP, diastolic blood pressure; FPG, fasting plasma glucose; AST, aspartate aminotransferase; ALT, alanine aminotransferase; γGTP, gamma-glutamyl transferase; TC, total cholesterol; TG, triglyceride; TyG index, triglyceride glucose index; HDL-C, high-density lipoprotein cholesterol; LDL-C, low-density lipoprotein cholesterol; eGFR, estimated glomerular filtration rate; ABI, ankle-brachial index; baPWV, brachial to ankle pulse wave velocity; CI, confidence interval. (*p < 0.05; **p < 0.01; ***p < 0.001)

## Discussion

After controlling for covariables, the results of this population-based cross-sectional study showed the highest tertile (above 8.57) of the TyG index had a positive and linear correlation with the baPWV as a marker of arterial stiffness in a general Japanese population. The results were stable in subgroups according to sex, age, BMI, smoking, alcohol, exercise, menopause, TC, LDLC, eGFR, and fatty liver. The study findings provide evidence for the involvement of IR in subclinical atherosclerosis in a Japanese population. Still, the exact causal relationship remains unknown, and determining the exact relationship will require longitudinal studies.

The relationship between the TyG index and baPWV in the Japanese population was mainly studied in this study. The standards of TG and FPG in different countries are different, and the normal ranges are different [28–30]. Furthermore, the TG and FPG are affected by genetics, the environment, and living habits [31–33]. Therefore, the relationship between TyG and baPWV can vary among countries. Future studies should examine and compare this relationship among countries. Studies showed relationships between TyG and baPWV in Chinese [16, 18] and Koreans [34]. Of note, the causality relationship between TyG and baPWV has not been determined yet [17]. Although it is true that the present study reported an association already observed in Chinese and Koreans, it now showed the relation in Japanese as well. Being an insular nation, Japan has genetics distinct from continental Asia.

Lee et al. [34] indicated that the optimal cut-off value of the TyG index to define insulin resistance has not yet been standardized. This study found that the highest tertile (> 8.57) of the TyG index had a positive and linear association with the baPWV. It is consistent with the 8.55 cut-off value of the TyG index reported by Lu et al. [35].

Previous studies have also reported the relationship between the TyG index and baPWV. A cross-sectional study found that the TyG index was not significantly associated with baPWV [16]. On the other hand, another cross-sectional study found that the TyG index was independently and positively associated with baPWV in hypertensive patients [18]. Furthermore, a cross-sectional study of Japanese participants revealed that the TyG index correlated with HOMA-IR, especially with increased high baPWV in multivariable linear regression analyses [19]. Moreover, the associations were stronger in women than in men based on their study [19]. Glucose levels are used for the calculation of the TyG index and HOMA-IR. HOMA-IR uses insulin levels, while TyG uses the TG levels. Although indirect, there is a relationship between insulin and TG levels. Indeed, insulin levels are elevated in insulin resistance because the β-cells have to compensate for the decreased peripheral insulin sensitivity. In insulin resistance, the liver also overproduces very low-density lipoproteins because of increased free fatty acid released by the adipocytes, leading to high triglyceride levels [36, 37]. Several factors can be responsible for arterial stiffness, including decreased elastin and increased collagen contents in the arterial wall, abnormal regulation of arterial smooth muscle tone, and advanced glycosylation end-products (AGE) [38, 39], and the accumulation of AGE is accelerated in insulin resistance states [40]. Furthermore, insulin resistance is associated with endothelial dysfunction [39–41], and the high insulin resistance levels stimulate the proliferation of the arterial smooth muscle cells [42]. Therefore, insulin resistance participates in arterial stiffness.

In this study, the subgroup analyses of the association between the TyG index and baPWV showed that both males and females had a significant steady relationship. After adjusting for BMI, age, TC, LDL-C, eGFR, and not having fatty liver disease, the subgroup analyses of this study revealed that they were significantly related to the TyG index and baPWV, which might be because our study had a relatively small sample size. This was somewhat similar to the report by Zhao et al., where the TyG index was significantly associated with arterial stiffness for macrovascular arteriosclerosis and CKD for microvascular arteriosclerosis. These associations were revealed after adjusting for age, sex, BMI, waist circumference, smoking habit, hypertension, family history of premature CVD, diabetes, HDL-C, LDL-C, insulin therapy, and statin therapy [43]. Keeping the relatively smaller sample size in mind compared to the report by Zhao et al., the TyG index and baPWV association were still somewhat similar.

Stepwise regression analysis showed that TC and LDL-C might influence the correlation between the TyG index and baPWV [34]. This study divided TC and LDL-C into two parts for subgroup analysis, which showed that TC, LDL-C, and TyG index had no interaction effect on baPWV. Therefore, the positive association between the TyG index and baPWV was robust. Furthermore, Baydar et al. stated that insulin resistance (IR) is an important risk factor in accelerating atherosclerosis. The TyG index, considered a predictor of IR, is associated with subclinical atherosclerosis [44]. Chiu et al. also reported a significant association between a high TyG index and atherosclerosis [16].

Our study suggested that the TyG index indicated the risk of carotid atherosclerosis, and IR has a relationship with subclinical atherosclerosis, but the exact nature of this relationship cannot be determined by the present study. Therefore, considering the TyG index a predictor of IR, a significant portion of morbidity and mortality related to CVDs could be prevented by identifying and preventing atherosclerosis. Besides, there are several advantages of this study, which are: (1) This study followed strict inclusion criteria, which were (a) excluded patients with symptomatic coronary artery disease (CAD), (b) included a broad age range of patients between 24 and 84 years and (c) ABI < 0.95. (2) This study reported the relationship between the TyG index and baPWV in a general Japanese population. (3) This study analyzed the TyG index as both continuous and categorical variables to reduce the contingency and increase the robustness of the result. (4) The likelihood ratio tests and hierarchical linear regression model were applied in subgroup analysis to confirm modifications and interactions.

This study has several limitations: (1) This is a cross-sectional study; therefore, causal relationships could not be determined. (2) Previous studies demonstrated a close relationship between HOMA-IR and the TyG index [45]. However, the analysis in this current study did not include the HOMA-IR index. The reason being is that the TyG index is more cost-effective and easier to calculate in routine clinical practice, as previously reported [46]. Thus, we plan to conduct the HOMA-IR index in future research on this topic to overcome this shortcoming.

## Conclusions

To summarize, the results of this study showed an independent association between the TyG index and arterial stiffness, as measured by baPWV, in a relatively healthy Japanese population after adjusting for conventional factors. These results suggest that IR is involved in subclinical atherosclerosis and might be an important target for preventing major CV disease. Still, the determination of the exact nature of this involvement will require longitudinal studies.

## Data Availability

The original data are freely downloaded at http://www.Datadryad.org/.

## Abbreviations

TyG index: The triglyceride/glucose index
baPWV: The brachial-ankle pulse wave velocity
NAGALA: NAFLD in Gifu Area, Longitudinal Analysis
adj OR: Adjusted odds ratio
95% CI: 95% confidence interval
CVDs: Cardiovascular diseases
IR: Insulin resistance
HOMA-IR: The homeostasis model assessment of insulin resistance
BMI: Body mass index
SBP: Systolic blood pressure
DBP: Diastolic blood pressure
ALT: Alanine aminotransferase
AST: Aspartate aminotransferase
γGTP: Gamma-glutamyl transferase
FPG: Fasting plasma glucose
UA: Uric acid
TC: Total cholesterol
TG: Triglyceride
HDL-C: High-density lipoprotein cholesterol
LDL-C: Low-density lipoprotein cholesterol
eGFR: Estimated glomerular filtration rate
ABI: Ankle-brachial index
HBV: Hepatitis B virus
HCV: Hepatitis C virus

## References

[1] Lopez D, Asher CR (2017). Congenital absence of the pericardium. Prog Cardiovasc Dis.

[2] Kalayinia S, Arjmand F, Maleki M, Malakootian M, Singh CP (2021). MicroRNAs: roles in cardiovascular development and disease. Cardiovasc Pathol.

[3] Tang Dalin, Li Zhi-Yong, Gijsen Frank, Giddens Don P (2015). Cardiovascular diseases and vulnerable plaques: data, modeling, predictions and clinical applications. BioMed Eng OnLine.

[4] Kim HL, Kim SH (2019). Pulse wave velocity in atherosclerosis. Front Cardiovasc Med.

[5] Zieman SJ, Melenovsky V, Kass DA (2005). Mechanisms, pathophysiology, and therapy of arterial stiffness. Arterioscler Thromb Vasc Biol.

[6] Chirinos JA, Segers P, Hughes T, Townsend R (2019). Large-artery stiffness in health and disease: JACC state-of-the-art review. J Am Coll Cardiol.

[7] Toth PP (2014). Insulin resistance, small LDL particles, and risk for atherosclerotic disease. Curr Vasc Pharmacol.

[8] Simental-Mendía LE, Rodríguez-Morán M, Guerrero-Romero F (2008). The product of fasting glucose and triglycerides as surrogate for identifying insulin resistance in apparently healthy subjects. Metab Syndr Relat Disord.

[9] Mohd Nor NS, Lee S, Bacha F, Tfayli H, Arslanian S (2016). Triglyceride glucose index as a surrogate measure of insulin sensitivity in obese adolescents with normoglycemia, prediabetes, and type 2 diabetes mellitus: comparison with the hyperinsulinemic-euglycemic clamp. Pediatr Diabetes.

[10] Kang B, Yang Y, Lee EY, Yang HK, Kim H-S, Lim S-Y, Lee J-H, Lee S-S, Suh B-K, Yoon K-H (2017). Triglycerides/glucose index is a useful surrogate marker of insulin resistance among adolescents. Int J Obes.

[11] Munakata M (2014). Brachial-ankle pulse wave velocity in the measurement of arterial stiffness: recent evidence and clinical applications. Curr Hypertens Rev.

[12] Huang J, Chen Z, Yuan J, Zhang C, Chen H, Wu W, Chen Z, Liu Y, Zheng M, Chen S (2019). Association between body mass index (BMI) and brachial-ankle pulse wave velocity (baPWV) in males with hypertension: a community-based cross-section study in North China. Med Sci Monit.

[13] Liu Y, Zhao P, Cheng M, Yu L, Cheng Z, Fan L, Chen C (2018). AST to ALT ratio and arterial stiffness in non-fatty liver Japanese population:a secondary analysis based on a cross-sectional study. Lipids Health Dis.

[14] Shouling W, Jin C, Li S, Zheng X, Zhang X, Cui L, Gao X (2019). Aging, arterial stiffness, and blood pressure association in Chinese adults. Hypertension.

[15] Zheng M, Zhang X, Chen S, Song Y, Zhao Q, Gao X, Wu S (2020). Arterial stiffness preceding diabetes: a longitudinal study. Circ Res.

[16] Chiu TH, Tsai HJ, Chiou HC, Wu PY, Huang JC, Chen SC (2021). A high triglyceride-glucose index is associated with left ventricular dysfunction and atherosclerosis. Int J Med Sci.

[17] Alizargar J, Bai CH, Hsieh NC, Wu SV (2020). Use of the triglyceride-glucose index (TyG) in cardiovascular disease patients. Cardiovasc Diabetol.

[18] Li M, Zhan A, Huang X, Hu L, Zhou W, Wang T, Zhu L, Bao H, Cheng X (2020). Positive association between triglyceride glucose index and arterial stiffness in hypertensive patients: the China H-type hypertension registry study. Cardiovasc Diabetol.

[19] Nakagomi A, Sunami Y, Kawasaki Y, Fujisawa T, Kobayashi Y (2020). Sex difference in the association between surrogate markers of insulin resistance and arterial stiffness. J Diabetes Complicat.

[20] Lambrinoudaki I, Kazani MV, Armeni E, Georgiopoulos G, Tampakis K, Rizos D, Augoulea A, Kaparos G, Alexandrou A, Stamatelopoulos K (2018). The TyG Index as a marker of subclinical atherosclerosis and arterial stiffness in lean and overweight postmenopausal women. Heart Lung Circ.

[21] Fukuda T, Hamaguchi M, Kojima T, Ohshima Y, Ohbora A, Kato T, Nakamura N, Fukui M (2014). Association between serum γ-glutamyltranspeptidase and atherosclerosis: a population-based cross-sectional study. BMJ Open.

[22] Hashimoto Y, Hamaguchi M, Kojima T, Ohshima Y, Ohbora A, Kato T, Nakamura N, Fukui M (2015). Modest alcohol consumption reduces the incidence of fatty liver in men: a population-based large-scale cohort study. J Gastroenterol Hepatol.

[23] Ryu S, Chang Y, Kim DI, Kim WS, Suh BS (2007). gamma-Glutamyltransferase as a predictor of chronic kidney disease in nonhypertensive and nondiabetic Korean men. Clin Chem.

[24] Hamaguchi M, Kojima T, Itoh Y, Harano Y, Fujii K, Nakajima T, Kato T, Takeda N, Okuda J, Ida K (2007). The severity of ultrasonographic findings in nonalcoholic fatty liver disease reflects the metabolic syndrome and visceral fat accumulation. Am J Gastroenterol.

[25] Yamashina A, Tomiyama H, Takeda K, Tsuda H, Arai T, Hirose K, Koji Y, Hori S, Yamamoto Y (2002). Validity, reproducibility, and clinical significance of noninvasive brachial-ankle pulse wave velocity measurement. Hypertens Res.

[26] Matsuo S, Imai E, Horio M, Yasuda Y, Tomita K, Nitta K, Yamagata K, Tomino Y, Yokoyama H, Hishida A (2009). Revised equations for estimated GFR from serum creatinine in Japan. Am J Kidney Dis.

[27] Higashioka M, Sakata S, Honda T, Hata J, Yoshida D, Hirakawa Y, Shibata M, Goto K, Kitazono T, Osawa H (2020). Small dense low-density lipoprotein cholesterol and the risk of coronary heart disease in a Japanese community. J Atheroscler Thromb.

[28] Kaur V, Verma M, Kaur A, Gupta S, Singh K (2012). To establish the reference intervals of lipid profile in punjab. Indian J Clin Biochem.

[29] De Henauw S, Michels N, Vyncke K, Hebestreit A, Russo P, Intemann T, Peplies J, Fraterman A, Eiben G, de Lorgeril M (2005). Blood lipids among young children in Europe: results from the European IDEFICS study. Int J Obes.

[30] Nielsen TRH, Lausten-Thomsen U, Fonvig CE, Bojsoe C, Pedersen L, Bratholm PS, Hansen T, Pedersen O, Holm JC (2017). Dyslipidemia and reference values for fasting plasma lipid concentrations in Danish/North-European White children and adolescents. BMC Pediatr.

[31] Oh B, Sung J, Chun S (2019). Potentially modifiable blood triglyceride levels by the control of conventional risk factors. Lipids Health Dis.

[32] Justesen JM, Andersson EA, Allin KH, Sandholt CH, Jorgensen T, Linneberg A, Jorgensen ME, Hansen T, Pedersen O, Grarup N (2016). Increasing insulin resistance accentuates the effect of triglyceride-associated loci on serum triglycerides during 5 years. J Lipid Res.

[33] Kolb H, Martin S (2017). Environmental/lifestyle factors in the pathogenesis and prevention of type 2 diabetes. BMC Med.

[34] Lee SB, Ahn CW, Lee BK, Kang S, Nam JS, You JH, Kim MJ, Kim MK, Park JS (2018). Association between triglyceride glucose index and arterial stiffness in Korean adults. Cardiovasc Diabetol.

[35] Lu YW, Chang CC, Chou RH, Tsai YL, Liu LK, Chen LK, Huang PH, Lin SJ (2021). Gender difference in the association between TyG index and subclinical atherosclerosis: results from the I-Lan longitudinal aging study. Cardiovasc Diabetol.

[36] Ginsberg HN, Zhang YL, Hernandez-Ono A (2005). Regulation of plasma triglycerides in insulin resistance and diabetes. Arch Med Res.

[37] Ma M, Liu H, Yu J, He S, Li P, Ma C, Zhang H, Xu L, Ping F, Li W (2020). Triglyceride is independently correlated with insulin resistance and islet beta cell function: a study in population with different glucose and lipid metabolism states. Lipids Health Dis.

[38] Lakatta EG, Levy D (2003). Arterial and cardiac aging: major shareholders in cardiovascular disease enterprises: Part I: aging arteries: a "set up" for vascular disease. Circulation.

[39] Sengstock DM, Vaitkevicius PV, Supiano MA (2005). Arterial stiffness is related to insulin resistance in nondiabetic hypertensive older adults. J Clin Endocrinol Metab.

[40] Singh R, Barden A, Mori T, Beilin L (2001). Advanced glycation end-products: a review. Diabetologia.

[41] Arcaro G, Cretti A, Balzano S, Lechi A, Muggeo M, Bonora E, Bonadonna RC (2002). Insulin causes endothelial dysfunction in humans: sites and mechanisms. Circulation.

[42] Indolfi C, Torella D, Cavuto L, Davalli AM, Coppola C, Esposito G, Carriero MV, Rapacciuolo A, Di Lorenzo E, Stabile E (2001). Effects of balloon injury on neointimal hyperplasia in streptozotocin-induced diabetes and in hyperinsulinemic nondiabetic pancreatic islet-transplanted rats. Circulation.

[43] Zhao S, Yu S, Chi C, Fan X, Tang J, Ji H, Teliewubai J, Zhang Y, Xu Y (2019). Association between macro- and microvascular damage and the triglyceride glucose index in community-dwelling elderly individuals: the Northern Shanghai study. Cardiovasc Diabetol.

[44] Baydar O, Kilic A, Okcuoglu J, Apaydin Z, Can MM (2021). The Triglyceride-glucose index, a predictor of insulin resistance is associated with subclinical atherosclerosis. Angiology.

[45] Du T, Yuan G, Zhang M, Zhou X, Sun X, Yu X (2014). Clinical usefulness of lipid ratios, visceral adiposity indicators, and the triglycerides and glucose index as risk markers of insulin resistance. Cardiovasc Diabetol.

[46] Liu EQ, Weng YP, Zhou AM, Zeng CL (2020). Association between Triglyceride-glucose index and type 2 diabetes mellitus in the Japanese population: a secondary analysis of a retrospective cohort study. Biomed Res Int.

